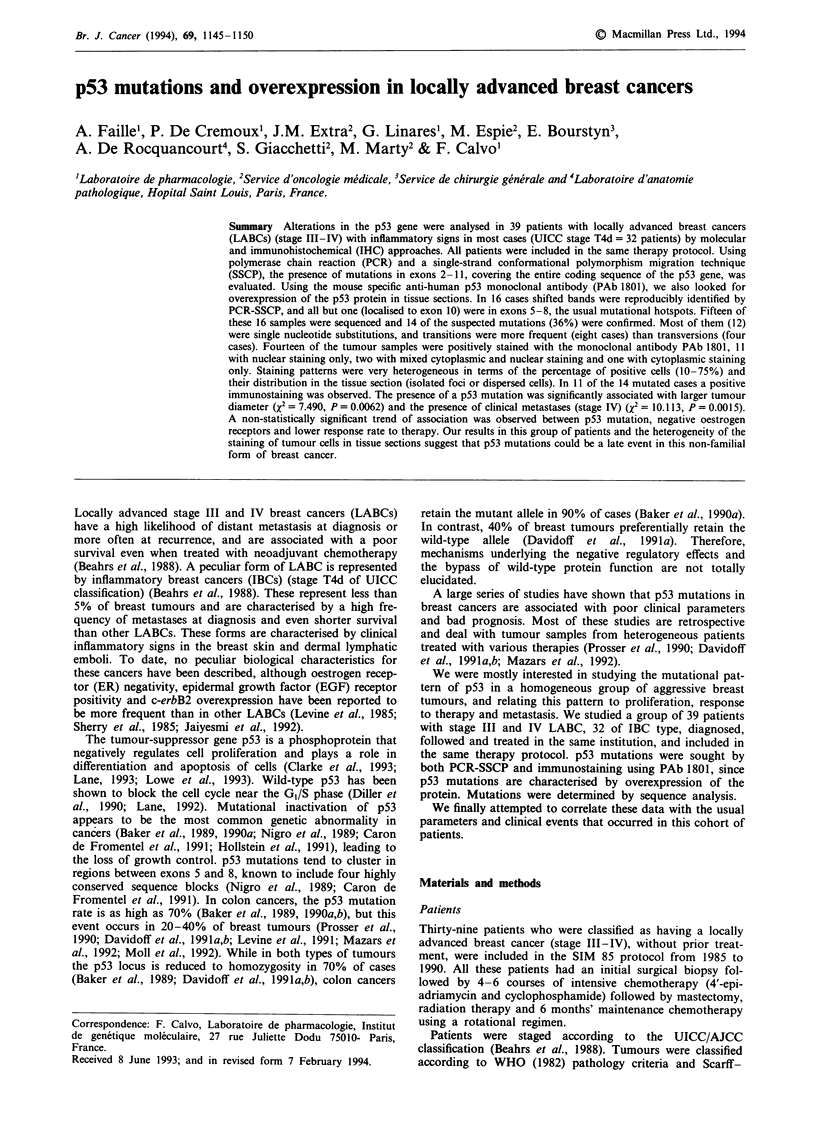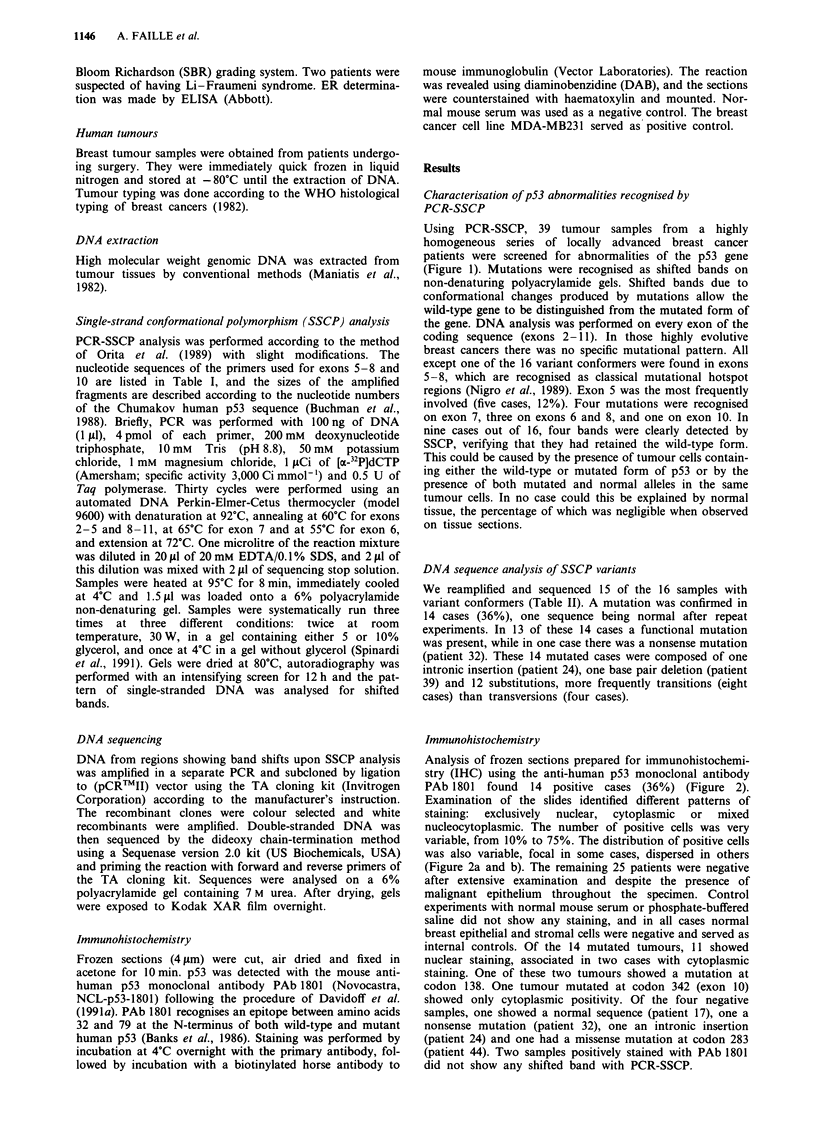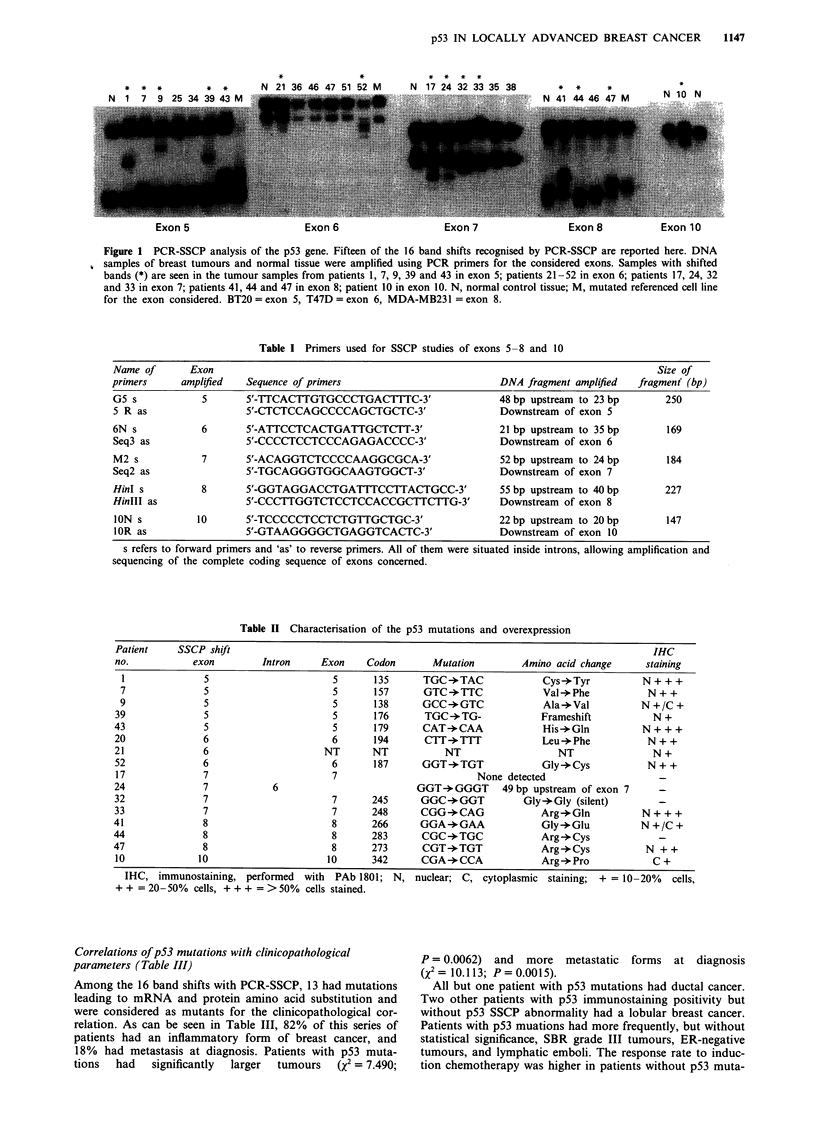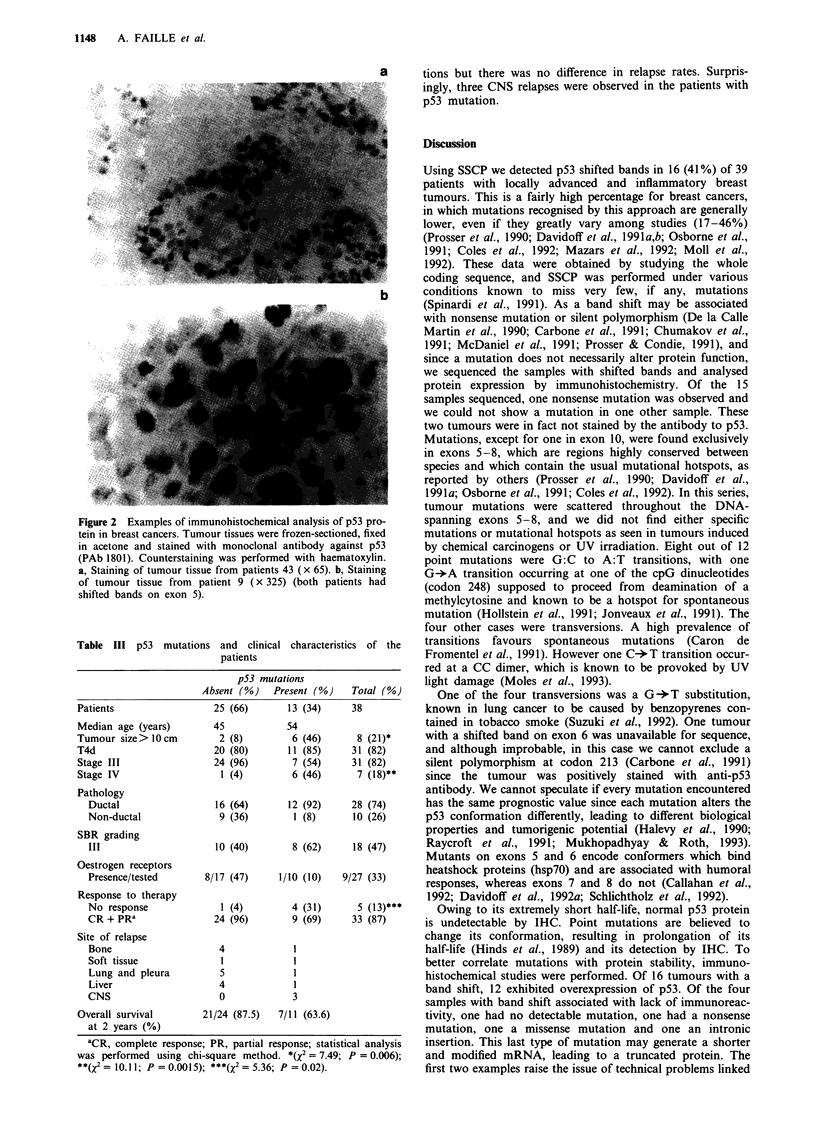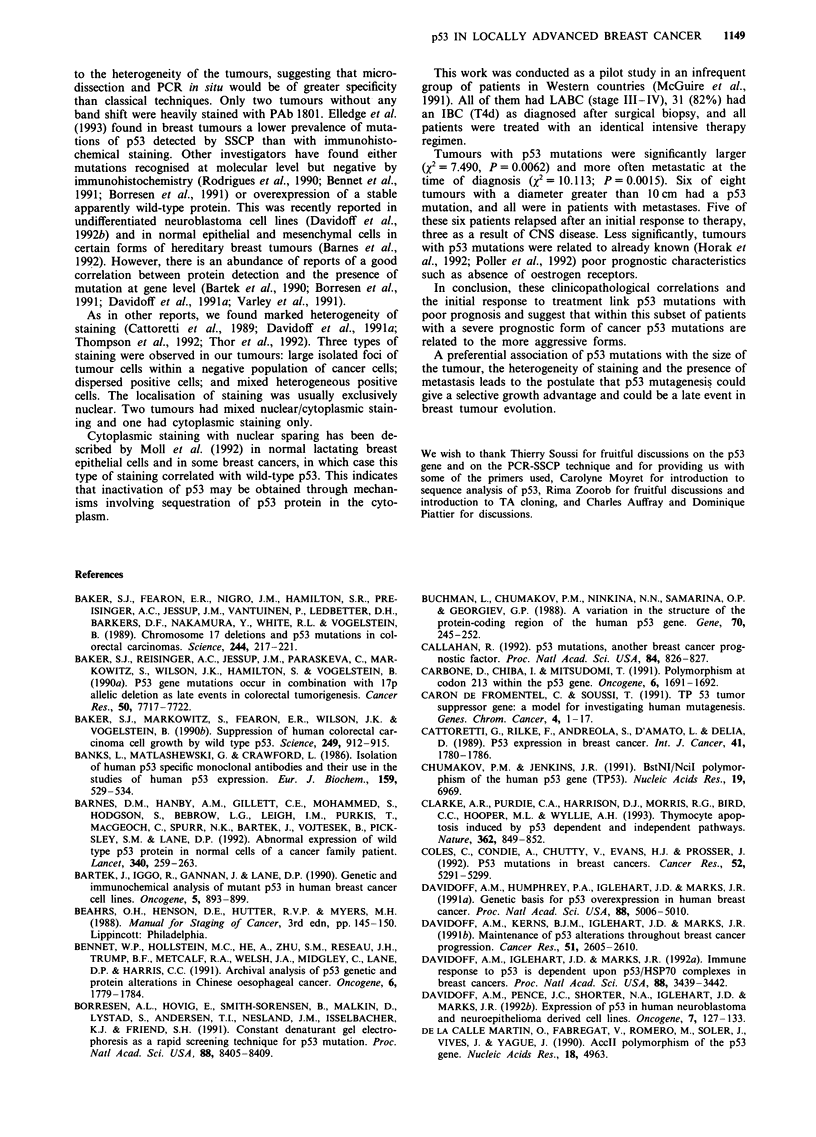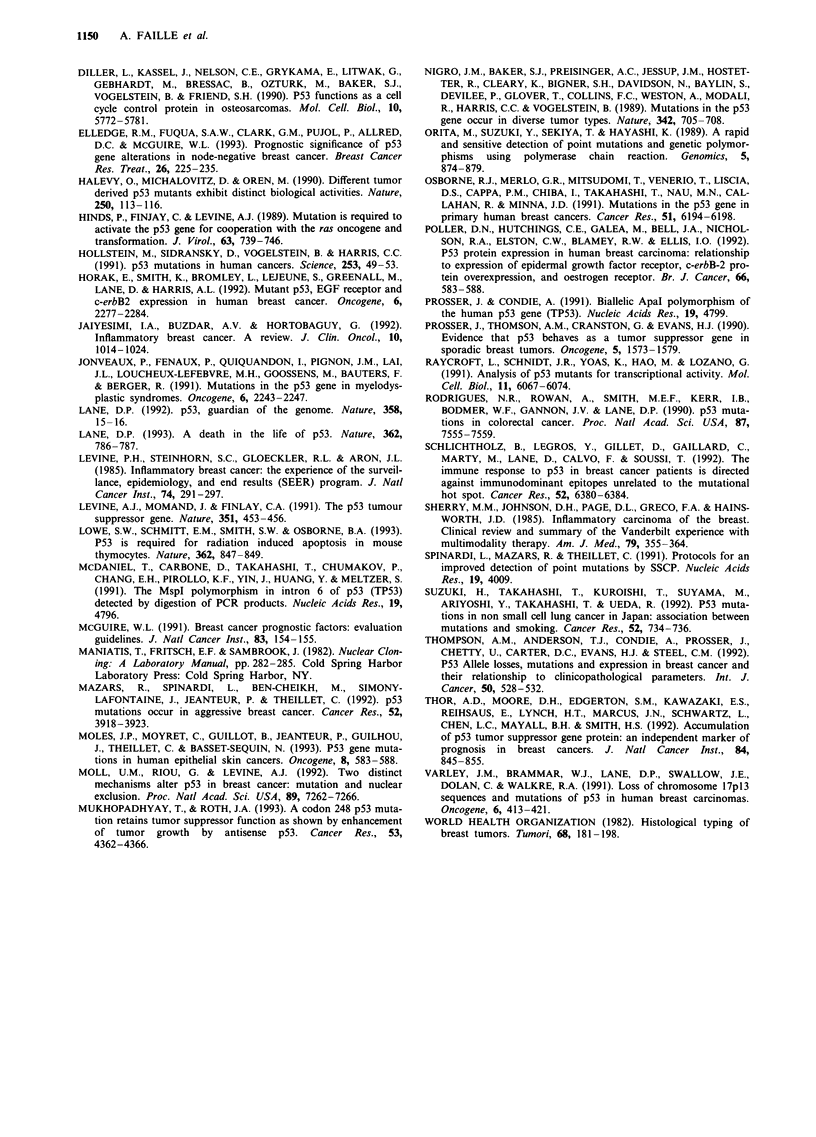# p53 mutations and overexpression in locally advanced breast cancers.

**DOI:** 10.1038/bjc.1994.225

**Published:** 1994-06

**Authors:** A. Faille, P. De Cremoux, J. M. Extra, G. Linares, M. Espie, E. Bourstyn, A. De Rocquancourt, S. Giacchetti, M. Marty, F. Calvo

**Affiliations:** Laboratoire de pharmacologie, Hospital Saint Louis, Paris, France.

## Abstract

**Images:**


Br. J. Cancer (1994), 69, 1145–1150 © Macmillan Press Ltd., 1994

### p53 mutations and overexpression in locally advanced breast cancers

A. Faille[1], P. De Cremoux[1], J.M. Extra[2], G. Linares[1], M. Espie[2], E. Bourstyn[3], A. De Rocquancourt[4], S. Giacchetti[2], M. Marty[2] & F. Calvo[1]

[1]*Laboratoire de pharmacologie,* [2]*Service d'oncologie médicale,* [3]*Service de chirurgie générale and* [4]*Laboratoire d'anatomie pathologique, Hopital Saint Louis, Paris, France.*

**Summary** Alterations in the p53 gene were analysed in 39 patients with locally advanced breast cancers (LABCs) (stage III–IV) with inflammatory signs in most cases (UICC stage T4d = 32 patients) by molecular and immunohistochemical (IHC) approaches. All patients were included in the same therapy protocol. Using polymerase chain reaction (PCR) and a single-strand conformational polymorphism migration technique (SSCP), the presence of mutations in exons 2–11, covering the entire coding sequence of the p53 gene, was evaluated. Using the mouse specific anti-human p53 monoclonal antibody (PAb 1801), we also looked for overexpression of the p53 protein in tissue sections. In 16 cases shifted bands were reproducibly identified by PCR-SSCP, and all but one (localised to exon 10) were in exons 5–8, the usual mutational hotspots. Fifteen of these 16 samples were sequenced and 14 of the suspected mutations (36%) were confirmed. Most of them (12) were single nucleotide substitutions, and transitions were more frequent (eight cases) than transversions (four cases). Fourteen of the tumour samples were positively stained with the monoclonal antibody PAb 1801, 11 with nuclear staining only, two with mixed cytoplasmic and nuclear staining and one with cytoplasmic staining only. Staining patterns were very heterogeneous in terms of the percentage of positive cells (10–75%) and their distribution in the tissue section (isolated foci or dispersed cells). In 11 of the 14 mutated cases a positive immunostaining was observed. The presence of a p53 mutation was significantly associated with larger tumour diameter ($\chi^2 = 7.490$, $P = 0.0062$) and the presence of clinical metastases (stage IV) ($\chi^2 = 10.113$, $P = 0.0015$). A non-statistically significant trend of association was observed between p53 mutation, negative oestrogen receptors and lower response rate to therapy. Our results in this group of patients and the heterogeneity of the staining of tumour cells in tissue sections suggest that p53 mutations could be a late event in this non-familial form of breast cancer.

Locally advanced stage III and IV breast cancers (LABCs) have a high likelihood of distant metastasis at diagnosis or more often at recurrence, and are associated with a poor survival even when treated with neoadjuvant chemotherapy (Beahrs *et al.*, 1988). A peculiar form of LABC is represented by inflammatory breast cancers (IBCs) (stage T4d of UICC classification) (Beahrs *et al.*, 1988). These represent less than 5% of breast tumours and are characterised by a high frequency of metastases at diagnosis and even shorter survival than other LABCs. These forms are characterised by clinical inflammatory signs in the breast skin and dermal lymphatic emboli. To date, no peculiar biological characteristics for these cancers have been described, although oestrogen receptor (ER) negativity, epidermal growth factor (EGF) receptor positivity and c-*erb*B2 overexpression have been reported to be more frequent than in other LABCs (Levine *et al.*, 1985; Sherry *et al.*, 1985; Jaiyesmi *et al.*, 1992).

The tumour-suppressor gene p53 is a phosphoprotein that negatively regulates cell proliferation and plays a role in differentiation and apoptosis of cells (Clarke *et al.*, 1993; Lane, 1993; Lowe *et al.*, 1993). Wild-type p53 has been shown to block the cell cycle near the $G_1/S$ phase (Diller *et al.*, 1990; Lane, 1992). Mutational inactivation of p53 appears to be the most common genetic abnormality in cancers (Baker *et al.*, 1989, 1990*a*; Nigro *et al.*, 1989; Caron de Fromentel *et al.*, 1991; Hollstein *et al.*, 1991), leading to the loss of growth control. p53 mutations tend to cluster in regions between exons 5 and 8, known to include four highly conserved sequence blocks (Nigro *et al.*, 1989; Caron de Fromentel *et al.*, 1991). In colon cancers, the p53 mutation rate is as high as 70% (Baker *et al.*, 1989, 1990*a,b*), but this event occurs in 20–40% of breast tumours (Prosser *et al.*, 1990; Davidoff *et al.*, 1991*a,b*; Levine *et al.*, 1991; Mazars *et al.*, 1992; Moll *et al.*, 1992). While in both types of tumours the p53 locus is reduced to homozygosity in 70% of cases (Baker *et al.*, 1989; Davidoff *et al.*, 1991*a,b*), colon cancers retain the mutant allele in 90% of cases (Baker *et al.*, 1990*a*). In contrast, 40% of breast tumours preferentially retain the wild-type allele (Davidoff *et al.*, 1991*a*). Therefore, mechanisms underlying the negative regulatory effects and the bypass of wild-type protein function are not totally elucidated.

A large series of studies have shown that p53 mutations in breast cancers are associated with poor clinical parameters and bad prognosis. Most of these studies are retrospective and deal with tumour samples from heterogeneous patients treated with various therapies (Prosser *et al.*, 1990; Davidoff *et al.*, 1991*a,b*; Mazars *et al.*, 1992).

We were mostly interested in studying the mutational pattern of p53 in a homogeneous group of aggressive breast tumours, and relating this pattern to proliferation, response to therapy and metastasis. We studied a group of 39 patients with stage III and IV LABC, 32 of IBC type, diagnosed, followed and treated in the same institution, and included in the same therapy protocol. p53 mutations were sought by both PCR-SSCP and immunostaining using PAb 1801, since p53 mutations are characterised by overexpression of the protein. Mutations were determined by sequence analysis.

We finally attempted to correlate these data with the usual parameters and clinical events that occurred in this cohort of patients.

### Materials and methods

### *Patients*

Thirty-nine patients who were classified as having a locally advanced breast cancer (stage III–IV), without prior treatment, were included in the SIM 85 protocol from 1985 to 1990. All these patients had an initial surgical biopsy followed by 4–6 courses of intensive chemotherapy (4′-epi-adriamycin and cyclophosphamide) followed by mastectomy, radiation therapy and 6 months' maintenance chemotherapy using a rotational regimen.

Patients were staged according to the UICC/AJCC classification (Beahrs *et al.*, 1988). Tumours were classified according to WHO (1982) pathology criteria and Scarff–

Correspondence: F. Calvo, Laboratoire de pharmacologie, Institut de genétique moléculaire, 27 rue Juliette Dodu 75010- Paris, France.
Received 8 June 1993; and in revised form 7 February 1994.

Bloom Richardson (SBR) grading system. Two patients were suspected of having Li-Fraumeni syndrome. ER determination was made by ELISA (Abbott).

*Human tumours*

Breast tumour samples were obtained from patients undergoing surgery. They were immediately quick frozen in liquid nitrogen and stored at −80°C until the extraction of DNA. Tumour typing was done according to the WHO histological typing of breast cancers (1982).

*DNA extraction*

High molecular weight genomic DNA was extracted from tumour tissues by conventional methods (Maniatis et al., 1982).

*Single-strand conformational polymorphism (SSCP) analysis*

PCR-SSCP analysis was performed according to the method of Orita et al. (1989) with slight modifications. The nucleotide sequences of the primers used for exons 5–8 and 10 are listed in Table I, and the sizes of the amplified fragments are described according to the nucleotide numbers of the Chumakov human p53 sequence (Buchman et al., 1988). Briefly, PCR was performed with 100 ng of DNA (1 μl), 4 pmol of each primer, 200 mM deoxynucleotide triphosphate, 10 mM Tris (pH 8.8), 50 mM potassium chloride, 1 mM magnesium chloride, 1 μCi of [α-$^{32}$P]dCTP (Amersham; specific activity 3,000 Ci mmol$^{-1}$) and 0.5 U of *Taq* polymerase. Thirty cycles were performed using an automated DNA Perkin-Elmer-Cetus thermocycler (model 9600) with denaturation at 92°C, annealing at 60°C for exons 2–5 and 8–11, at 65°C for exon 7 and at 55°C for exon 6, and extension at 72°C. One microlitre of the reaction mixture was diluted in 20 μl of 20 mM EDTA/0.1% SDS, and 2 μl of this dilution was mixed with 2 μl of sequencing stop solution. Samples were heated at 95°C for 8 min, immediately cooled at 4°C and 1.5 μl was loaded onto a 6% polyacrylamide non-denaturing gel. Samples were systematically run three times at three different conditions: twice at room temperature, 30 W, in a gel containing either 5 or 10% glycerol, and once at 4°C in a gel without glycerol (Spinardi et al., 1991). Gels were dried at 80°C, autoradiography was performed with an intensifying screen for 12 h and the pattern of single-stranded DNA was analysed for shifted bands.

*DNA sequencing*

DNA from regions showing band shifts upon SSCP analysis was amplified in a separate PCR and subcloned by ligation to (pCR™II) vector using the TA cloning kit (Invitrogen Corporation) according to the manufacturer's instruction. The recombinant clones were colour selected and white recombinants were amplified. Double-stranded DNA was then sequenced by the dideoxy chain-termination method using a Sequenase version 2.0 kit (US Biochemicals, USA) and priming the reaction with forward and reverse primers of the TA cloning kit. Sequences were analysed on a 6% polyacrylamide gel containing 7 M urea. After drying, gels were exposed to Kodak XAR film overnight.

*Immunohistochemistry*

Frozen sections (4 μm) were cut, air dried and fixed in acetone for 10 min. p53 was detected with the mouse anti-human p53 monoclonal antibody PAb 1801 (Novocastra, NCL-p53-1801) following the procedure of Davidoff et al. (1991*a*). PAb 1801 recognises an epitope between amino acids 32 and 79 at the N-terminus of both wild-type and mutant human p53 (Banks et al., 1986). Staining was performed by incubation at 4°C overnight with the primary antibody, followed by incubation with a biotinylated horse antibody to mouse immunoglobulin (Vector Laboratories). The reaction was revealed using diaminobenzidine (DAB), and the sections were counterstained with haematoxylin and mounted. Normal mouse serum was used as a negative control. The breast cancer cell line MDA-MB231 served as positive control.

### Results

*Characterisation of p53 abnormalities recognised by PCR-SSCP*

Using PCR-SSCP, 39 tumour samples from a highly homogeneous series of locally advanced breast cancer patients were screened for abnormalities of the p53 gene (Figure 1). Mutations were recognised as shifted bands on non-denaturing polyacrylamide gels. Shifted bands due to conformational changes produced by mutations allow the wild-type gene to be distinguished from the mutated form of the gene. DNA analysis was performed on every exon of the coding sequence (exons 2–11). In those highly evolutive breast cancers there was no specific mutational pattern. All except one of the 16 variant conformers were found in exons 5–8, which are recognised as classical mutational hotspot regions (Nigro et al., 1989). Exon 5 was the most frequently involved (five cases, 12%). Four mutations were recognised on exon 7, three on exons 6 and 8, and one on exon 10. In nine cases out of 16, four bands were clearly detected by SSCP, verifying that they had retained the wild-type form. This could be caused by the presence of tumour cells containing either the wild-type or mutated form of p53 or by the presence of both mutated and normal alleles in the same tumour cells. In no case could this be explained by normal tissue, the percentage of which was negligible when observed on tissue sections.

*DNA sequence analysis of SSCP variants*

We reamplified and sequenced 15 of the 16 samples with variant conformers (Table II). A mutation was confirmed in 14 cases (36%), one sequence being normal after repeat experiments. In 13 of these 14 cases a functional mutation was present, while in one case there was a nonsense mutation (patient 32). These 14 mutated cases were composed of one intronic insertion (patient 24), one base pair deletion (patient 39) and 12 substitutions, more frequently transitions (eight cases) than transversions (four cases).

*Immunohistochemistry*

Analysis of frozen sections prepared for immunohistochemistry (IHC) using the anti-human p53 monoclonal antibody PAb 1801 found 14 positive cases (36%) (Figure 2). Examination of the slides identified different patterns of staining: exclusively nuclear, cytoplasmic or mixed nucleocytoplasmic. The number of positive cells was very variable, from 10% to 75%. The distribution of positive cells was also variable, focal in some cases, dispersed in others (Figure 2a and b). The remaining 25 patients were negative after extensive examination and despite the presence of malignant epithelium throughout the specimen. Control experiments with normal mouse serum or phosphate-buffered saline did not show any staining, and in all cases normal breast epithelial and stromal cells were negative and served as internal controls. Of the 14 mutated tumours, 11 showed nuclear staining, associated in two cases with cytoplasmic staining. One of these two tumours showed a mutation at codon 138. One tumour mutated at codon 342 (exon 10) showed only cytoplasmic positivity. Of the four negative samples, one showed a normal sequence (patient 17), one a nonsense mutation (patient 32), one an intronic insertion (patient 24) and one had a missense mutation at codon 283 (patient 44). Two samples positively stained with PAb 1801 did not show any shifted band with PCR-SSCP.

**Figure 1** PCR-SSCP analysis of the p53 gene. Fifteen of the 16 band shifts recognised by PCR-SSCP are reported here. DNA samples of breast tumours and normal tissue were amplified using PCR primers for the considered exons. Samples with shifted bands (*) are seen in the tumour samples from patients 1, 7, 9, 39 and 43 in exon 5; patients 21–52 in exon 6; patients 17, 24, 32 and 33 in exon 7; patients 41, 44 and 47 in exon 8; patient 10 in exon 10. N, normal control tissue; M, mutated referenced cell line for the exon considered. BT20 = exon 5, T47D = exon 6, MDA-MB231 = exon 8.

**Table I** Primers used for SSCP studies of exons 5–8 and 10

| *Name of primers* | *Exon amplified* | *Sequence of primers* | *DNA fragment amplified* | *Size of fragment (bp)* |
|---|---|---|---|---|
| G5 s | 5 | 5′-TTCACTTGTGCCCTGACTTTC-3′ | 48 bp upstream to 23 bp | 250 |
| 5 R as | | 5′-CTCTCCAGCCCCAGCTGCTC-3′ | Downstream of exon 5 | |
| 6N s | 6 | 5′-ATTCCTCACTGATTGCTCTT-3′ | 21 bp upstream to 35 bp | 169 |
| Seq3 as | | 5′-CCCCTCCTCCCAGAGACCCC-3′ | Downstream of exon 6 | |
| M2 s | 7 | 5′-ACAGGTCTCCCCAAGGCGCA-3′ | 52 bp upstream to 24 bp | 184 |
| Seq2 as | | 5′-TGCAGGGTGGCAAGTGGCT-3′ | Downstream of exon 7 | |
| *Hin*I s | 8 | 5′-GGTAGGACCTGATTTCCTTACTGCC-3′ | 55 bp upstream to 40 bp | 227 |
| *Hin*III as | | 5′-CCCTTGGTCTCCTCCACCGCTTCTTG-3′ | Downstream of exon 8 | |
| 10N s | 10 | 5′-TCCCCCTCCTCTGTTGCTGC-3′ | 22 bp upstream to 20 bp | 147 |
| 10R as | | 5′-GTAAGGGGCTGAGGTCACTC-3′ | Downstream of exon 10 | |

s refers to forward primers and 'as' to reverse primers. All of them were situated inside introns, allowing amplification and sequencing of the complete coding sequence of exons concerned.

**Table II** Characterisation of the p53 mutations and overexpression

| *Patient no.* | *SSCP shift exon* | *Intron* | *Exon* | *Codon* | *Mutation* | *Amino acid change* | *IHC staining* |
|---|---|---|---|---|---|---|---|
| 1 | 5 | | 5 | 135 | TGC→TAC | Cys→Tyr | N+++ |
| 7 | 5 | | 5 | 157 | GTC→TTC | Val→Phe | N++ |
| 9 | 5 | | 5 | 138 | GCC→GTC | Ala→Val | N+/C+ |
| 39 | 5 | | 5 | 176 | TGC→TG- | Frameshift | N+ |
| 43 | 5 | | 5 | 179 | CAT→CAA | His→Gln | N+++ |
| 20 | 6 | | 6 | 194 | CTT→TTT | Leu→Phe | N++ |
| 21 | 6 | | NT | NT | NT | NT | N+ |
| 52 | 6 | | 6 | 187 | GGT→TGT | Gly→Cys | N++ |
| 17 | 7 | | 7 | | None detected | | – |
| 24 | 7 | 6 | | | GGT→GGGT | 49 bp upstream of exon 7 | – |
| 32 | 7 | | 7 | 245 | GGC→GGT | Gly→Gly (silent) | – |
| 33 | 7 | | 7 | 248 | CGG→CAG | Arg→Gln | N+++ |
| 41 | 8 | | 8 | 266 | GGA→GAA | Gly→Glu | N+/C+ |
| 44 | 8 | | 8 | 283 | CGC→TGC | Arg→Cys | – |
| 47 | 8 | | 8 | 273 | CGT→TGT | Arg→Cys | N ++ |
| 10 | 10 | | 10 | 342 | CGA→CCA | Arg→Pro | C+ |

IHC, immunostaining, performed with PAb 1801; N, nuclear; C, cytoplasmic staining; + = 10–20% cells, ++ = 20–50% cells, +++ = >50% cells stained.

*Correlations of p53 mutations with clinicopathological parameters (Table III)*

Among the 16 band shifts with PCR-SSCP, 13 had mutations leading to mRNA and protein amino acid substitution and were considered as mutants for the clinicopathological correlation. As can be seen in Table III, 82% of this series of patients had an inflammatory form of breast cancer, and 18% had metastasis at diagnosis. Patients with p53 mutations had significantly larger tumours ($\chi^2 = 7.490$; $P = 0.0062$) and more metastatic forms at diagnosis ($\chi^2 = 10.113$; $P = 0.0015$).

All but one patient with p53 mutations had ductal cancer. Two other patients with p53 immunostaining positivity but without p53 SSCP abnormality had a lobular breast cancer. Patients with p53 muations had more frequently, but without statistical significance, SBR grade III tumours, ER-negative tumours, and lymphatic emboli. The response rate to induction chemotherapy was higher in patients without p53 muta-

Figure 2 Examples of immunohistochemical analysis of p53 protein in breast cancers. Tumour tissues were frozen-sectioned, fixed in acetone and stained with monoclonal antibody against p53 (PAb 1801). Counterstaining was performed with haematoxylin. a, Staining of tumour tissue from patients 43 (× 65). b, Staining of tumour tissue from patient 9 (× 325) (both patients had shifted bands on exon 5).

**Table III** p53 mutations and clinical characteristics of the patients

| | *p53 mutations* Absent (%) | Present (%) | Total (%) |
|---|---|---|---|
| Patients | 25 (66) | 13 (34) | 38 |
| Median age (years) | 45 | 54 | |
| Tumour size > 10 cm | 2 (8) | 6 (46) | 8 (21)* |
| T4d | 20 (80) | 11 (85) | 31 (82) |
| Stage III | 24 (96) | 7 (54) | 31 (82) |
| Stage IV | 1 (4) | 6 (46) | 7 (18)** |
| Pathology | | | |
| Ductal | 16 (64) | 12 (92) | 28 (74) |
| Non-ductal | 9 (36) | 1 (8) | 10 (26) |
| SBR grading | | | |
| III | 10 (40) | 8 (62) | 18 (47) |
| Oestrogen receptors | | | |
| Presence/tested | 8/17 (47) | 1/10 (10) | 9/27 (33) |
| Response to therapy | | | |
| No response | 1 (4) | 4 (31) | 5 (13)*** |
| CR + PR[a] | 24 (96) | 9 (69) | 33 (87) |
| Site of relapse | | | |
| Bone | 4 | 1 | |
| Soft tissue | 1 | 1 | |
| Lung and pleura | 5 | 1 | |
| Liver | 4 | 1 | |
| CNS | 0 | 3 | |
| Overall survival at 2 years (%) | 21/24 (87.5) | 7/11 (63.6) | |

[a]CR, complete response; PR, partial response; statistical analysis was performed using chi-square method. *($\chi^2 = 7.49$; $P = 0.006$); **($\chi^2 = 10.11$; $P = 0.0015$); ***($\chi^2 = 5.36$; $P = 0.02$).

tions but there was no difference in relapse rates. Surprisingly, three CNS relapses were observed in the patients with p53 mutation.

### Discussion

Using SSCP we detected p53 shifted bands in 16 (41%) of 39 patients with locally advanced and inflammatory breast tumours. This is a fairly high percentage for breast cancers, in which mutations recognised by this approach are generally lower, even if they greatly vary among studies (17–46%) (Prosser *et al.*, 1990; Davidoff *et al.*, 1991*a*,*b*; Osborne *et al.*, 1991; Coles *et al.*, 1992; Mazars *et al.*, 1992; Moll *et al.*, 1992). These data were obtained by studying the whole coding sequence, and SSCP was performed under various conditions known to miss very few, if any, mutations (Spinardi *et al.*, 1991). As a band shift may be associated with nonsense mutation or silent polymorphism (De la Calle Martin *et al.*, 1990; Carbone *et al.*, 1991; Chumakov *et al.*, 1991; McDaniel *et al.*, 1991; Prosser & Condie, 1991), and since a mutation does not necessarily alter protein function, we sequenced the samples with shifted bands and analysed protein expression by immunohistochemistry. Of the 15 samples sequenced, one nonsense mutation was observed and we could not show a mutation in one other sample. These two tumours were in fact not stained by the antibody to p53. Mutations, except for one in exon 10, were found exclusively in exons 5–8, which are regions highly conserved between species and which contain the usual mutational hotspots, as reported by others (Prosser *et al.*, 1990; Davidoff *et al.*, 1991*a*; Osborne *et al.*, 1991; Coles *et al.*, 1992). In this series, tumour mutations were scattered throughout the DNA-spanning exons 5–8, and we did not find either specific mutations or mutational hotspots as seen in tumours induced by chemical carcinogens or UV irradiation. Eight out of 12 point mutations were G:C to A:T transitions, with one G→A transition occurring at one of the cpG dinucleotides (codon 248) supposed to proceed from deamination of a methylcytosine and known to be a hotspot for spontaneous mutation (Hollstein *et al.*, 1991; Jonveaux *et al.*, 1991). The four other cases were transversions. A high prevalence of transitions favours spontaneous mutations (Caron de Fromentel *et al.*, 1991). However one C→T transition occurred at a CC dimer, which is known to be provoked by UV light damage (Moles *et al.*, 1993).

One of the four transversions was a G→T substitution, known in lung cancer to be caused by benzopyrenes contained in tobacco smoke (Suzuki *et al.*, 1992). One tumour with a shifted band on exon 6 was unavailable for sequence, and although improbable, in this case we cannot exclude a silent polymorphism at codon 213 (Carbone *et al.*, 1991) since the tumour was positively stained with anti-p53 antibody. We cannot speculate if every mutation encountered has the same prognostic value since each mutation alters the p53 conformation differently, leading to different biological properties and tumorigenic potential (Halevy *et al.*, 1990; Raycroft *et al.*, 1991; Mukhopadhyay & Roth, 1993). Mutants on exons 5 and 6 encode conformers which bind heatshock proteins (hsp70) and are associated with humoral responses, whereas exons 7 and 8 do not (Callahan *et al.*, 1992; Davidoff *et al.*, 1992*a*; Schlichtholz *et al.*, 1992).

Owing to its extremely short half-life, normal p53 protein is undetectable by IHC. Point mutations are believed to change its conformation, resulting in prolongation of its half-life (Hinds *et al.*, 1989) and its detection by IHC. To better correlate mutations with protein stability, immunohistochemical studies were performed. Of 16 tumours with a band shift, 12 exhibited overexpression of p53. Of the four samples with band shift associated with lack of immunoreactivity, one had no detectable mutation, one had a nonsense mutation, one a missense mutation and one an intronic insertion. This last type of mutation may generate a shorter and modified mRNA, leading to a truncated protein. The first two examples raise the issue of technical problems linked to the heterogeneity of the tumours, suggesting that microdissection and PCR *in situ* would be of greater specificity than classical techniques. Only two tumours without any band shift were heavily stained with PAb 1801. Elledge *et al.* (1993) found in breast tumours a lower prevalence of mutations of p53 detected by SSCP than with immunohistochemical staining. Other investigators have found either mutations recognised at molecular level but negative by immunohistochemistry (Rodrigues *et al.*, 1990; Bennet *et al.*, 1991; Borresen *et al.*, 1991) or overexpression of a stable apparently wild-type protein. This was recently reported in undifferentiated neuroblastoma cell lines (Davidoff *et al.*, 1992*b*) and in normal epithelial and mesenchymal cells in certain forms of hereditary breast tumours (Barnes *et al.*, 1992). However, there is an abundance of reports of a good correlation between protein detection and the presence of mutation at gene level (Bartek *et al.*, 1990; Borresen *et al.*, 1991; Davidoff *et al.*, 1991*a*; Varley *et al.*, 1991).

As in other reports, we found marked heterogeneity of staining (Cattoretti *et al.*, 1989; Davidoff *et al.*, 1991*a*; Thompson *et al.*, 1992; Thor *et al.*, 1992). Three types of staining were observed in our tumours: large isolated foci of tumour cells within a negative population of cancer cells; dispersed positive cells; and mixed heterogeneous positive cells. The localisation of staining was usually exclusively nuclear. Two tumours had mixed nuclear/cytoplasmic staining and one had cytoplasmic staining only.

Cytoplasmic staining with nuclear sparing has been described by Moll *et al.* (1992) in normal lactating breast epithelial cells and in some breast cancers, in which case this type of staining correlated with wild-type p53. This indicates that inactivation of p53 may be obtained through mechanisms involving sequestration of p53 protein in the cytoplasm.

This work was conducted as a pilot study in an infrequent group of patients in Western countries (McGuire *et al.*, 1991). All of them had LABC (stage III–IV), 31 (82%) had an IBC (T4d) as diagnosed after surgical biopsy, and all patients were treated with an identical intensive therapy regimen.

Tumours with p53 mutations were significantly larger ($\chi^2 = 7.490$, $P = 0.0062$) and more often metastatic at the time of diagnosis ($\chi^2 = 10.113$; $P = 0.0015$). Six of eight tumours with a diameter greater than 10 cm had a p53 mutation, and all were in patients with metastases. Five of these six patients relapsed after an initial response to therapy, three as a result of CNS disease. Less significantly, tumours with p53 mutations were related to already known (Horak *et al.*, 1992; Poller *et al.*, 1992) poor prognostic characteristics such as absence of oestrogen receptors.

In conclusion, these clinicopathological correlations and the initial response to treatment link p53 mutations with poor prognosis and suggest that within this subset of patients with a severe prognostic form of cancer p53 mutations are related to the more aggressive forms.

A preferential association of p53 mutations with the size of the tumour, the heterogeneity of staining and the presence of metastasis leads to the postulate that p53 mutagenesis could give a selective growth advantage and could be a late event in breast tumour evolution.

We wish to thank Thierry Soussi for fruitful discussions on the p53 gene and on the PCR-SSCP technique and for providing us with some of the primers used, Carolyne Moyret for introduction to sequence analysis of p53, Rima Zoorob for fruitful discussions and introduction to TA cloning, and Charles Auffray and Dominique Piattier for discussions.

### References

BAKER, S.J., FEARON, E.R., NIGRO, J.M., HAMILTON, S.R., PREISINGER, A.C., JESSUP, J.M., VANTUINEN, P., LEDBETTER, D.H., BARKERS, D.F., NAKAMURA, Y., WHITE, R.L. & VOGELSTEIN, B. (1989). Chromosome 17 deletions and p53 mutations in colorectal carcinomas. *Science*, **244**, 217–221.

BAKER, S.J., REISINGER, A.C., JESSUP, J.M., PARASKEVA, C., MARKOWITZ, S., WILSON, J.K., HAMILTON, S. & VOGELSTEIN, B. (1990*a*). P53 gene mutations occur in combination with 17p allelic deletion as late events in colorectal tumorigenesis. *Cancer Res.*, **50**, 7717–7722.

BAKER, S.J., MARKOWITZ, S., FEARON, E.R., WILSON, J.K. & VOGELSTEIN, B. (1990*b*). Suppression of human colorectal carcinoma cell growth by wild type p53. *Science*, **249**, 912–915.

BANKS, L., MATLASHEWSKI, G. & CRAWFORD, L. (1986). Isolation of human p53 specific monoclonal antibodies and their use in the studies of human p53 expression. *Eur. J. Biochem.*, **159**, 529–534.

BARNES, D.M., HANBY, A.M., GILLETT, C.E., MOHAMMED, S., HODGSON, S., BEBROW, L.G., LEIGH, I.M., PURKIS, T., MACGEOCH, C., SPURR, N.K., BARTEK, J., VOJTESEK, B., PICKSLEY, S.M. & LANE, D.P. (1992). Abnormal expression of wild type p53 protein in normal cells of a cancer family patient. *Lancet*, **340**, 259–263.

BARTEK, J., IGGO, R., GANNAN, J. & LANE, D.P. (1990). Genetic and immunochemical analysis of mutant p53 in human breast cancer cell lines. *Oncogene*, **5**, 893–899.

BEAHRS, O.H., HENSON, D.E., HUTTER, R.V.P. & MYERS, M.H. (1988). *Manual for Staging of Cancer*, 3rd edn, pp. 145–150. Lippincott: Philadelphia.

BENNET, W.P., HOLLSTEIN, M.C., HE, A., ZHU, S.M., RESEAU, J.H., TRUMP, B.F., METCALF, R.A., WELSH, J.A., MIDGLEY, C., LANE, D.P. & HARRIS, C.C. (1991). Archival analysis of p53 genetic and protein alterations in Chinese oesophageal cancer. *Oncogene*, **6**, 1779–1784.

BORRESEN, A.L., HOVIG, E., SMITH-SORENSEN, B., MALKIN, D., LYSTAD, S., ANDERSEN, T.I., NESLAND, J.M., ISSELBACHER, K.J. & FRIEND, S.H. (1991). Constant denaturant gel electrophoresis as a rapid screening technique for p53 mutation. *Proc. Natl Acad. Sci. USA*, **88**, 8405–8409.

BUCHMAN, L., CHUMAKOV, P.M., NINKINA, N.N., SAMARINA, O.P. & GEORGIEV, G.P. (1988). A variation in the structure of the protein-coding region of the human p53 gene. *Gene*, **70**, 245–252.

CALLAHAN, R. (1992). p53 mutations, another breast cancer prognostic factor. *Proc. Natl Acad. Sci. USA*, **84**, 826–827.

CARBONE, D., CHIBA, I. & MITSUDOMI, T. (1991). Polymorphism at codon 213 within the p53 gene. *Oncogene*, **6**, 1691–1692.

CARON DE FROMENTEL, C. & SOUSSI, T. (1991). TP 53 tumor suppressor gene: a model for investigating human mutagenesis. *Genes. Chrom. Cancer*, **4**, 1–17.

CATTORETTI, G., RILKE, F., ANDREOLA, S., D'AMATO, L. & DELIA, D. (1989). P53 expression in breast cancer. *Int. J. Cancer*, **41**, 1780–1786.

CHUMAKOV, P.M. & JENKINS, J.R. (1991). BstNI/NciI polymorphism of the human p53 gene (TP53). *Nucleic Acids Res.*, **19**, 6969.

CLARKE, A.R., PURDIE, C.A., HARRISON, D.J., MORRIS, R.G., BIRD, C.C., HOOPER, M.L. & WYLLIE, A.H. (1993). Thymocyte apoptosis induced by p53 dependent and independent pathways. *Nature*, **362**, 849–852.

COLES, C., CONDIE, A., CHUTTY, V., EVANS, H.J. & PROSSER, J. (1992). P53 mutations in breast cancers. *Cancer Res.*, **52**, 5291–5299.

DAVIDOFF, A.M., HUMPHREY, P.A., IGLEHART, J.D. & MARKS, J.R. (1991*a*). Genetic basis for p53 overexpression in human breast cancer. *Proc. Natl Acad. Sci. USA*, **88**, 5006–5010.

DAVIDOFF, A.M., KERNS, B.J.M., IGLEHART, J.D. & MARKS, J.R. (1991*b*). Maintenance of p53 alterations throughout breast cancer progression. *Cancer Res.*, **51**, 2605–2610.

DAVIDOFF, A.M., IGLEHART, J.D. & MARKS, J.R. (1992*a*). Immune response to p53 is dependent upon p53/HSP70 complexes in breast cancers. *Proc. Natl Acad. Sci. USA*, **88**, 3439–3442.

DAVIDOFF, A.M., PENCE, J.C., SHORTER, N.A., IGLEHART, J.D. & MARKS, J.R. (1992*b*). Expression of p53 in human neuroblastoma and neuroepithelioma derived cell lines. *Oncogene*, **7**, 127–133.

DE LA CALLE MARTIN, O., FABREGAT, V., ROMERO, M., SOLER, J., VIVES, J. & YAGUE, J. (1990). AccII polymorphism of the p53 gene. *Nucleic Acids Res.*, **18**, 4963.

DILLER, L., KASSEL, J., NELSON, C.E., GRYKAMA, E., LITWAK, G., GEBHARDT, M., BRESSAC, B., OZTURK, M., BAKER, S.J., VOGELSTEIN, B. & FRIEND, S.H. (1990). P53 functions as a cell cycle control protein in osteosarcomas. *Mol. Cell. Biol.*, **10**, 5772-5781.

ELLEDGE, R.M., FUQUA, S.A.W., CLARK, G.M., PUJOL, P., ALLRED, D.C. & MCGUIRE, W.L. (1993). Prognostic significance of p53 gene alterations in node-negative breast cancer. *Breast Cancer Res. Treat.*, **26**, 225-235.

HALEVY, O., MICHALOVITZ, D. & OREN, M. (1990). Different tumor derived p53 mutants exhibit distinct biological activities. *Nature*, **250**, 113-116.

HINDS, P., FINJAY, C. & LEVINE, A.J. (1989). Mutation is required to activate the p53 gene for cooperation with the *ras* oncogene and transformation. *J. Virol.*, **63**, 739-746.

HOLLSTEIN, M., SIDRANSKY, D., VOGELSTEIN, B. & HARRIS, C.C. (1991). p53 mutations in human cancers. *Science*, **253**, 49-53.

HORAK, E., SMITH, K., BROMLEY, L., LEJEUNE, S., GREENALL, M., LANE, D. & HARRIS, A.L. (1992). Mutant p53, EGF receptor and c-*erb*B2 expression in human breast cancer. *Oncogene*, **6**, 2277-2284.

JAIYESIMI, I.A., BUZDAR, A.V. & HORTOBAGUY, G. (1992). Inflammatory breast cancer. A review. *J. Clin. Oncol.*, **10**, 1014-1024.

JONVEAUX, P., FENAUX, P., QUIQUANDON, I., PIGNON, J.M., LAI, J.L., LOUCHEUX-LEFEBVRE, M.H., GOOSSENS, M., BAUTERS, F. & BERGER, R. (1991). Mutations in the p53 gene in myelodysplastic syndromes. *Oncogene*, **6**, 2243-2247.

LANE, D.P. (1992). p53, guardian of the genome. *Nature*, **358**, 15-16.

LANE, D.P. (1993). A death in the life of p53. *Nature*, **362**, 786-787.

LEVINE, P.H., STEINHORN, S.C., GLOECKLER, R.L. & ARON, J.L. (1985). Inflammatory breast cancer: the experience of the surveillance, epidemiology, and end results (SEER) program. *J. Natl Cancer Inst.*, **74**, 291-297.

LEVINE, A.J., MOMAND, J. & FINLAY, C.A. (1991). The p53 tumour suppressor gene. *Nature*, **351**, 453-456.

LOWE, S.W., SCHMITT, E.M., SMITH, S.W. & OSBORNE, B.A. (1993). P53 is required for radiation induced apoptosis in mouse thymocytes. *Nature*, **362**, 847-849.

MCDANIEL, T., CARBONE, D., TAKAHASHI, T., CHUMAKOV, P., CHANG, E.H., PIROLLO, K.F., YIN, J., HUANG, Y. & MELTZER, S. (1991). The MspI polymorphism in intron 6 of p53 (TP53) detected by digestion of PCR products. *Nucleic Acids Res.*, **19**, 4796.

MCGUIRE, W.L. (1991). Breast cancer prognostic factors: evaluation guidelines. *J. Natl Cancer Inst.*, **83**, 154-155.

MANIATIS, T., FRITSCH, E.F. & SAMBROOK, J. (1982). *Nuclear Cloning: A Laboratory Manual*, pp. 282-285. Cold Spring Harbor Laboratory Press: Cold Spring Harbor, NY.

MAZARS, R., SPINARDI, L., BEN-CHEIKH, M., SIMONY-LAFONTAINE, J., JEANTEUR, P. & THEILLET, C. (1992). p53 mutations occur in aggressive breast cancer. *Cancer Res.*, **52**, 3918-3923.

MOLES, J.P., MOYRET, C., GUILLOT, B., JEANTEUR, P., GUILHOU, J., THEILLET, C. & BASSET-SEQUIN, N. (1993). P53 gene mutations in human epithelial skin cancers. *Oncogene*, **8**, 583-588.

MOLL, U.M., RIOU, G. & LEVINE, A.J. (1992). Two distinct mechanisms alter p53 in breast cancer: mutation and nuclear exclusion. *Proc. Natl Acad. Sci. USA*, **89**, 7262-7266.

MUKHOPADHYAY, T., & ROTH, J.A. (1993). A codon 248 p53 mutation retains tumor suppressor function as shown by enhancement of tumor growth by antisense p53. *Cancer Res.*, **53**, 4362-4366.

NIGRO, J.M., BAKER, S.J., PREISINGER, A.C., JESSUP, J.M., HOSTETTER, R., CLEARY, K., BIGNER, S.H., DAVIDSON, N., BAYLIN, S., DEVILEE, P., GLOVER, T., COLLINS, F.C., WESTON, A., MODALI, R., HARRIS, C.C. & VOGELSTEIN, B. (1989). Mutations in the p53 gene occur in diverse tumor types. *Nature*, **342**, 705-708.

ORITA, M., SUZUKI, Y., SEKIYA, T. & HAYASHI, K. (1989). A rapid and sensitive detection of point mutations and genetic polymorphisms using polymerase chain reaction. *Genomics*, **5**, 874-879.

OSBORNE, R.J., MERLO, G.R., MITSUDOMI, T., VENERIO, T., LISCIA, D.S., CAPPA, P.M., CHIBA, I., TAKAHASHI, T., NAU, M.N., CALLAHAN, R. & MINNA, J.D. (1991). Mutations in the p53 gene in primary human breast cancers. *Cancer Res.*, **51**, 6194-6198.

POLLER, D.N., HUTCHINGS, C.E., GALEA, M., BELL, J.A., NICHOLSON, R.A., ELSTON, C.W., BLAMEY, R.W. & ELLIS, I.O. (1992). P53 protein expression in human breast carcinoma: relationship to expression of epidermal growth factor receptor, c-*erb*B-2 protein overexpression, and oestrogen receptor. *Br. J. Cancer*, **66**, 583-588.

PROSSER, J. & CONDIE, A. (1991). Biallelic ApaI polymorphism of the human p53 gene (TP53). *Nucleic Acids Res.*, **19**, 4799.

PROSSER, J., THOMSON, A.M., CRANSTON, G. & EVANS, H.J. (1990). Evidence that p53 behaves as a tumor suppressor gene in sporadic breast tumors. *Oncogene*, **5**, 1573-1579.

RAYCROFT, L., SCHNIDT, J.R., YOAS, K., HAO, M. & LOZANO, G. (1991). Analysis of p53 mutants for transcriptional activity. *Mol. Cell. Biol.*, **11**, 6067-6074.

RODRIGUES, N.R., ROWAN, A., SMITH, M.E.F., KERR, I.B., BODMER, W.F., GANNON, J.V. & LANE, D.P. (1990). p53 mutations in colorectal cancer. *Proc. Natl Acad. Sci. USA*, **87**, 7555-7559.

SCHLICHTHOLZ, B., LEGROS, Y., GILLET, D., GAILLARD, C., MARTY, M., LANE, D., CALVO, F. & SOUSSI, T. (1992). The immune response to p53 in breast cancer patients is directed against immunodominant epitopes unrelated to the mutational hot spot. *Cancer Res.*, **52**, 6380-6384.

SHERRY, M.M., JOHNSON, D.H., PAGE, D.L., GRECO, F.A. & HAINSWORTH, J.D. (1985). Inflammatory carcinoma of the breast. Clinical review and summary of the Vanderbilt experience with multimodality therapy. *Am. J. Med.*, **79**, 355-364.

SPINARDI, L., MAZARS, R. & THEILLET, C. (1991). Protocols for an improved detection of point mutations by SSCP. *Nucleic Acids Res.*, **19**, 4009.

SUZUKI, H., TAKAHASHI, T., KUROISHI, T., SUYAMA, M., ARIYOSHI, Y., TAKAHASHI, T. & UEDA, R. (1992). P53 mutations in non small cell lung cancer in Japan: association between mutations and smoking. *Cancer Res.*, **52**, 734-736.

THOMPSON, A.M., ANDERSON, T.J., CONDIE, A., PROSSER, J., CHETTY, U., CARTER, D.C., EVANS, H.J. & STEEL, C.M. (1992). P53 Allele losses, mutations and expression in breast cancer and their relationship to clinicopathological parameters. *Int. J. Cancer*, **50**, 528-532.

THOR, A.D., MOORE, D.H., EDGERTON, S.M., KAWAZAKI, E.S., REIHSAUS, E., LYNCH, H.T., MARCUS, J.N., SCHWARTZ, L., CHEN, L.C., MAYALL, B.H. & SMITH, H.S. (1992). Accumulation of p53 tumor suppressor gene protein: an independent marker of prognosis in breast cancers. *J. Natl Cancer Inst.*, **84**, 845-855.

VARLEY, J.M., BRAMMAR, W.J., LANE, D.P., SWALLOW, J.E., DOLAN, C. & WALKRE, R.A. (1991). Loss of chromosome 17p13 sequences and mutations of p53 in human breast carcinomas. *Oncogene*, **6**, 413-421.

WORLD HEALTH ORGANIZATION (1982). Histological typing of breast tumors. *Tumori*, **68**, 181-198.